# The Respiratory Microbiome in Chronic Hypersensitivity Pneumonitis Is Distinct from That of Idiopathic Pulmonary Fibrosis

**DOI:** 10.1164/rccm.202002-0460OC

**Published:** 2021-02-01

**Authors:** Rachele Invernizzi, Benjamin G. Wu, Joseph Barnett, Poonam Ghai, Shaun Kingston, Richard J. Hewitt, Johanna Feary, Yonghua Li, Felix Chua, Zhe Wu, Athol U. Wells, Peter M. George, Elisabetta A. Renzoni, Andrew G. Nicholson, Alexandra Rice, Anand Devaraj, Leopoldo N. Segal, Adam J. Byrne, Toby M. Maher, Clare M. Lloyd, Philip L. Molyneaux

**Affiliations:** ^1^ National Heart and Lung Institute, Imperial College London, London, United Kingdom; ^2^ Division of Pulmonary and Critical Care Medicine, New York University, New York, New York; and; ^3^ Royal Brompton Hospital, London, United Kingdom

**Keywords:** lung microbiota, 16S, fibrosis

## Abstract

**Rationale**: Chronic hypersensitivity pneumonitis (CHP) is a condition that arises after repeated exposure and sensitization to inhaled antigens. The lung microbiome is increasingly implicated in respiratory disease, but, to date, no study has investigated the composition of microbial communities in the lower airways in CHP.

**Objectives:** To characterize and compare the airway microbiome in subjects with CHP, subjects with idiopathic pulmonary fibrosis (IPF), and control subjects.

**Methods:** We prospectively recruited individuals with a CHP diagnosis (*n* = 110), individuals with an IPF diagnosis (*n* = 45), and control subjects (*n* = 28). Subjects underwent BAL and bacterial DNA was isolated, quantified by quantitative PCR and the 16S ribosomal RNA gene was sequenced to characterize the bacterial communities in the lower airways.

**Measurements and Main Results:** Distinct differences in the microbial profiles were evident in the lower airways of subjects with CHP and IPF. At the phylum level, the prevailing microbiota of both subjects with IPF and subjects with CHP included *Firmicutes*, *Bacteroidetes*, *Proteobacteria*, and *Actinobacteria*. However, in IPF, *Firmicutes* dominated, whereas the percentage of reads assigned to *Proteobacteria* in the same group was significantly lower than the percentage found in subjects with CHP. At the genus level, the *Staphylococcus* burden was increased in CHP, and *Actinomyces* and *Veillonella* burdens were increased in IPF. The lower airway bacterial burden in subjects with CHP was higher than that in control subjects but lower than that of those with IPF. In contrast to IPF, there was no association between bacterial burden and survival in CHP.

**Conclusions:** The microbial profile of the lower airways in subjects with CHP is distinct from that of IPF, and, notably, the bacterial burden in individuals with CHP fails to predict survival.

**At a Glance Commentary**

**Scientific Knowledge on the Subject**

Numerous studies of the respiratory microbiome in idiopathic pulmonary fibrosis (IPF) have reported associations between changes in the bacterial burden, microbial communities, disease progression, and mortality. Chronic hypersensitivity pneumonitis (CHP) is a pathogenically and prognostically distinct disorder compared with IPF but is still characterized phenotypically by irreversible fibrotic destruction of the lung parenchyma. Despite the phenotypic overlap, no study has examined whether the respiratory microbiota differs between individuals with these conditions.

**What This Study Adds to the Field**

Despite the phenotypic overlap between CHP and IPF, there are clear differences in the bacterial burden and microbial composition in the lower airways of patients with CHP compared with healthy subjects and patients with IPF. In CHP there are increased proportions of *Proteobacteria* and lower proportions of *Firmicutes* compared with subjects with IPF. A higher bacterial burden has been consistently demonstrated in the lower airways in IPF and is associated with an increased risk of mortality. However, the bacterial burden in subjects with CHP was far lower than in those with IPF and did not associate with survival. Our findings suggest that the observed alterations in the lung microbiome are disease specific and do not simply reflect the presence of fibrosis within the lung.

Hypersensitivity pneumonitis is an immune-mediated interstitial lung disease that develops in genetically susceptible individuals after repeated inhalation of organic antigens, including fungal, bacterial, animal, and insect proteins (1). It can be classified into acute and chronic forms, depending on the nature of the inciting antigen, the intensity and duration of exposure, host factors, and radiological findings (2). Chronic hypersensitivity pneumonitis (CHP) carries the most significant morbidity and mortality. It arises from protracted exposure to a triggering antigen, which initially elicits inflammation and ultimately evolves into irreversible and often progressive interstitial fibrosis (3, 4). As yet, there are no established international guidelines for the diagnosis of CHP, and it remains unclear why only some individuals exposed to recognized triggers develop the disease (5). This diagnostic uncertainty is further confounded in advanced disease because the clinical features of CHP and idiopathic pulmonary fibrosis (IPF) are often indistinguishable (6).

The existence of a microbiome in the healthy lung is generally accepted, and its potential role in respiratory pathology is increasingly appreciated (7–9).The advent of next-generation sequencing technologies has allowed direct and culture-independent sequence-based interrogation of microbial communities in both homeostatic and perturbed states (10, 11). Changes in the respiratory microbiome have been associated with the clinical, physiological, and therapeutic aspects of chronic lung diseases such as asthma, bronchiectasis, chronic obstructive pulmonary disease (COPD), and IPF (12–18). Studies of the respiratory microbiome in IPF have reported associations between changes in microbial communities and disease progression and mortality (16, 17, 19, 20). A higher bacterial burden has been reported during acute exacerbations of IPF (21, 22) and is consistently associated with an increased risk of mortality (16–18). Differences in bacterial community composition and diversity have also been correlated with disease outcomes, peripheral blood host-defense gene expression (19), and indices of alveolar inflammation (18). These observational studies support the hypothesis that the respiratory microbiome may be implicated in the pathogenesis of fibrotic lung disease (23, 24).

CHP and IPF are pathogenically and prognostically distinct disorders that are both characterized by irreversible fibrotic destruction of the lung parenchyma; we set out to test the hypothesis that observed alterations in the lung microbiome are disease specific and do not simply reflect the presence of fibrosis within the lung. In this study, we prospectively recruited individuals with CHP and IPF diagnoses as well as healthy control subjects to characterize the bacterial communities in the lower airways by 16S ribosomal (r)RNA gene sequencing. Despite the phenotypic overlap between CHP and IPF, we demonstrate that there are clear differences in patients with CHP in the microbial composition and bacterial burden of the lower airways and in survival, compared with healthy subjects and patients with IPF.

## Methods

### Study Design

Treatment-naive subjects were prospectively recruited at the Royal Brompton Hospital (London) between May 2014 and May 2018. A diagnosis of IPF was made after multidisciplinary team discussion, according to the latest international guidelines (25). A consensus diagnosis of CHP was made using multidisciplinary team discussion with integration of clinical, radiological, BAL, and pathological data (5, 26). Pulmonary function testing was performed, clinical measurements were recorded, and subjects underwent fiberoptic bronchoscopy with BAL via the oropharyngeal route as part of standard care according to a standard operating procedure (27). BAL cell profiling was performed by assessing leukocyte differential counts. Computed tomographic data were scored as previously described (28). Approval for the study was obtained from the local research ethics committee (15/SC/0101 and 15-LO-1399), and written informed consent was obtained from all subjects. The IPF cohort was not used in any prior studies. *See* the online supplement for further details.

### Bacterial DNA Isolation and 16S rRNA Amplicon Sequencing

Genomic DNA was extracted from BAL cell pellets as previously described, with minor modifications (29). Amplification of the V4 hypervariable region of the 16S rRNA gene was performed using the barcoded universal primer pair 515F/806R and sequenced using an Illumina MiSeq instrument (Illumina) to produce 150 base-paired reads. For full details, *see* the online supplement.

### Bioinformatics Analysis

Reads were analyzed using the Quantitative Insights into Microbial Ecology 2 (version 2018.8) bioinformatics pipeline (30). Bioinformatics processing included demultiplexing, denoising, removal of chimeric and short reads, 97% identity binning of reads into amplicon sequence variants (ASV) using Divisive Amplicon Denoising Algorithm 2 (31), and taxonomic annotation using the Greengenes database (32). Absolute ASV sequence counts were normalized to obtain the relative abundances of the microbiota in each sample. After abundance filtering, a rarefied data set was generated and used for downstream analyses. For details on processing and quality control, *see* the online supplement.

### 16S rRNA Gene Quantitative PCR

Triplicate 10-μl quantitative PCR (qPCR) reactions were set up containing 1 μl of bacterial DNA and 9 μl of Femto bacterial qPCR premix (Cambridge Bioscience). Each run contained a 10-fold dilution series of the *Vibrio natriegens DSM 759* gene cloned into a plasmid of known size and a nontemplate control (28). For a description of cycling parameters, *see* the Methods section of the online supplement.

### Statistical Analysis

All data analysis was performed in R (version 3.4.3; R Foundation for Statistical Computing) (33). Differences in BAL differential cell counts, bacterial burden, and taxonomic composition among subjects with CHP, subjects with IPF, and control subjects were statistically tested by using the Kruskal-Wallis test and the Dunn test for multiple comparisons, which was adjusted using the Bonferroni method. Univariate linear regressions between bacterial burden and BAL differential cell counts were performed and adjusted for age, sex, smoking status, and FVC using a multivariable linear regression model. Changes in microbial biodiversity and richness between subjects with CHP and subjects with IPF were evaluated using the Shannon and Chao1 α-diversity indexes and were tested using the Mann-Whitney test. The β-diversity of subjects with CHP and subjects with IPF was restricted to ASV that were present at >1% of the sample population, calculated using the Euclidean distance and visualized by principal component analysis (PCA), with the significance of differences in community composition being determined by permutational multivariate ANOVA adjusted for age, sex, smoking status, FVC (% predicted), and Dl_CO_ (% predicted). The linear discriminant analysis effect size was used to identify differentially abundant bacteria among the groups. To study CHP and IPF disease progression, a Kaplan-Meier curve was assessed using a multivariate Cox proportional-hazard model, adjusted for age, sex, smoking status, FVC (% predicted), and percentage of honeycombing. Survival time was defined as the time from diagnosis to death, loss to follow-up, or the end of the study period. In all analyses, a *P* value < 0.05 was considered statistically significant.

### Data Availability

Sequences are available via the National Center for Biotechnology Information Sequence Read Archive (accession number PRJNA609242). Codes and metadata used for analysis are available at https://github.com/molyneaux-lab.

## Results

### Subjects, Sampling, and Sequencing

One hundred ten subjects with CHP, 45 subjects with IPF, and 28 control subjects were enrolled in the study and underwent bronchoscopy (Table 1). The demographics demonstrate a number of expected differences based on the known sex, smoking, and clinical profiles of both IPF and CHP (34). Subjects with IPF were predominantly male (82%), with a mean ± SD age of 62 ± 19 years and moderately severe restrictive lung disease (FVC, 75% predicted ± 19% predicted; Hb-adjusted Dl_CO_, 44% predicted ± 17% predicted). There were more female subjects in the CHP cohort, and although their mean age (66 ± 9 years) and Dl_CO_ (44% predicted ± 16% predicted) were matched, they had a more preserved FVC compared with the IPF cohort (85% predicted ± 23% predicted). The median follow-up in the CHP cohort was 919 days, and the median follow-up in the IPF cohort was 744 days (not significant). Although alveolar macrophages predominated in the BAL cells of all groups, a mixed alveolitis with predominant neutrophilia was observed in subjects with IPF (18% ± 6% neutrophils, 12% ± 5% lymphocytes), whereas subjects with CHP demonstrated an elevated lymphocyte count (32% ± 15% lymphocytes). Radiologically, the subjects with CHP had more ground-glass and low-density lobules and less honeycombing than subjects with IPF. In keeping with the literature, over half (52%) of the patients with CHP had no obvious exposure history. The majority of exposures in the CHP cohort were mold-related (20%), avian (15.45%) or feather down–related (5.45%). In contrast, only eight patients with IPF had an obvious exposure history (17.7%) (Table 1). All 183 samples yielded genomic DNA amplicons and underwent sequencing of the V4 region of the 16S rRNA gene. After demultiplexing, denoising, quality control, and abundance filtering, 8,406,014 sequences were retained, representing 495 unique ASV across 183 samples (35). This final curated data set was used for all subsequent analyses.

**Table 1.** Baseline Characteristics of Study Subjects

Parameter	Control Subjects (*n* = *28*)	CHP (*n* = *110*)	IPF (*n* = *45*)	*P* Value*
Age, yr	55 ± 15	66 ± 9	62 ± 19	NS
Sex, F, *n* (%)	7 (25)	54 (49)	8 (18)	<0.001
Baseline PFT				
FVC, % predicted	94 ± 18	85 ± 23	75 ± 19	<0.01
FEV_1_, % predicted	73 ± 23	86 ± 22	78 ± 17	<0.05
Dl_CO_, % predicted	68 ± 32	44 ± 16	44 ± 17	NS
Smoking status^†^				
Nonsmoker, *n* (%)	7 (25)	56 (51)	15 (33)	—
Ex-smoker, *n* (%)	10 (36)	54 (49)	26 (58)	NS
Current smoker, *n* (%)	1 (4)	0 (0)	4 (9)	—
BAL cell differentials				
Macrophages, %	70 ± 16	62 ± 15	60 ± 8	NS
Neutrophils, %	3 ± 2	10 ± 9	18 ± 6	<0.0001
Lymphocytes, %	10 ± 6	32 ± 15	12 ± 5	<0.0001
Radiology^‡^				
Fibrosis present, *n* (%)	NA	98 (89)	44 (100)	<0.05
UIP, *n* (%)	NA	31 (28)	35 (78)	<0.001
Honeycombing, *n* (%)	NA	10 (9)	13 (29)	<0.01
GG extent %, median (IQR)	NA	10 (14)	5 (10)	<0.01
Low-density lobule extent, *n* (%)	NA	5 (12)	5 (8)	<0.05
Antigen exposure^§^				
Avian, *n* (%)	NA	17 (15)	1 (2)	<0.05
Feather down, *n* (%)	NA	6 (5)	1 (2)	NS
Mold, *n* (%)	NA	22 (20)	3 (7)	NS
Other, *n* (%)	NA	8 (7)	3 (7)	NS
None, *n* (%)	NA	57 (52)	37 (82)	<0.001

*Definition of abbreviations*: CHP = chronic hypersensitivity pneumonitis; GG = ground glass; IPF = idiopathic pulmonary fibrosis; IQR = interquartile range; NA = not applicable; NS = not significant; PFT = pulmonary function test; UIP = usual interstitial pneumonia.

Data are presented as the mean ± SD unless otherwise noted.

* *P* values were calculated by using the Mann-Whitney *U* test or the Fisher exact test between subjects with CHP and subjects with IPF.

^†^ Smoking status: statistical testing by nonsmoker versus ex/current smoker; data were available for 18 of 28 control subjects.

^‡^ Fibrosis was defined as the presence of traction bronchiectasis and/or honeycombing. UIP was defined as “definite” or “probable” UIP. Radiology data for low-density lobules and ground glass were scored to the nearest 5% on a lobar basis, with the lingua as a sixth lobe. The final score represents the mean of the lobar scores.

^§^ Antigen exposure: statistical testing by exposure versus no exposure.

### Bacterial Burden

**Figure 1.**
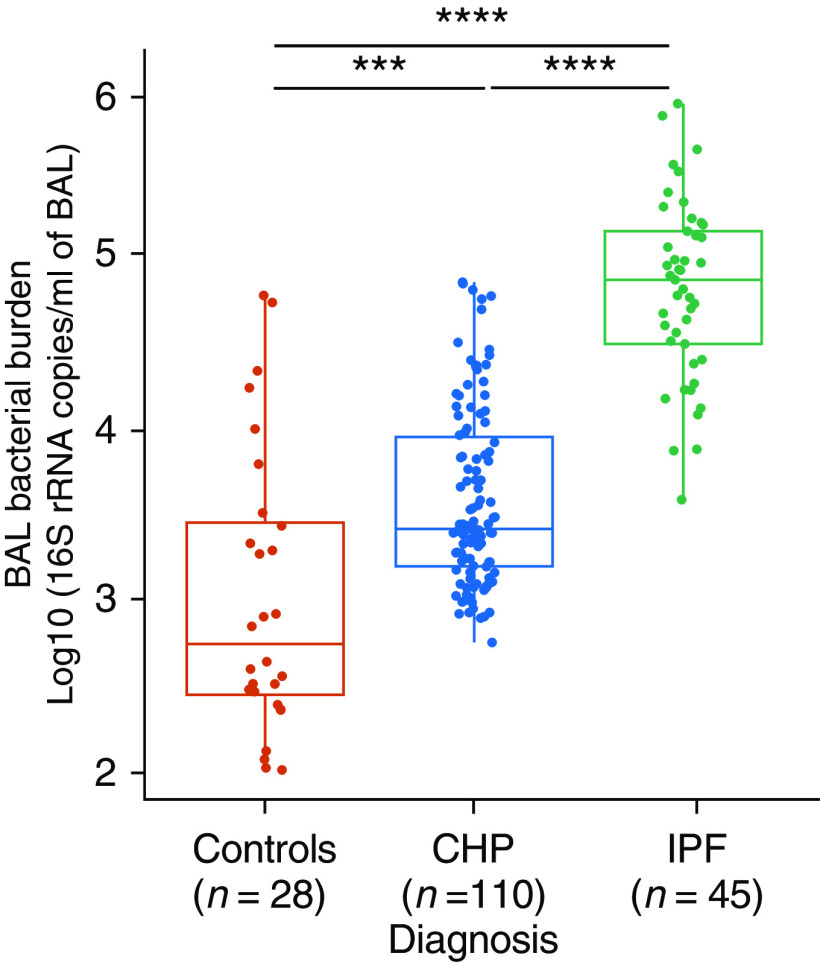
Bacterial burden in BAL fluid of control subjects, subjects with CHP, and subjects with IPF. The Kruskal-Wallis test with the Dunn test for multiple comparisons was used (*P* < 2.2 × 10^−16^). Data are presented as the median and interquartile range (***P < 0.0001 and ****P < 0.00001). The bacterial burden was calculated by using quantitative PCR and was expressed in log_10_ values (16S ribosomal RNA gene copies/ml of BAL fluid). CHP = chronic hypersensitivity pneumonitis; IPF = idiopathic pulmonary fibrosis.

The strongest and most robust microbial signature associated with fibrotic lung disease to date is that of the bacterial burden. We and others have already shown that IPF is characterized by an increased bacterial burden in BAL cells, which is associated with disease progression. We therefore initially set out to explore differences in the BAL bacterial burden among control subjects and subjects with CHP and IPF. On average, subjects with IPF had 1.54 × 10^5^ copies of the 16S rRNA gene per milliliter of BAL fluid, which was higher than the average copy number in control subjects (8.57 × 10^3^ copies/ml; *P* < 0.00001). Although subjects with CHP had a higher bacterial load (1.14 × 10^4^) compared with healthy control subjects (*P* < 0.0001), they had a significantly lower burden compared with subjects with IPF (*P* < 0.00001) (Figure 1). No correlation was found between the bacterial load and the BAL differential cell counts (*see* Figure E1 in the online supplement). The negative control samples yielded a bacterial burden close to or below the lower limit of qPCR quantification (100 copies/ml of BAL fluid) (Figure E2).

### Survival Analysis

**Figure 2.**
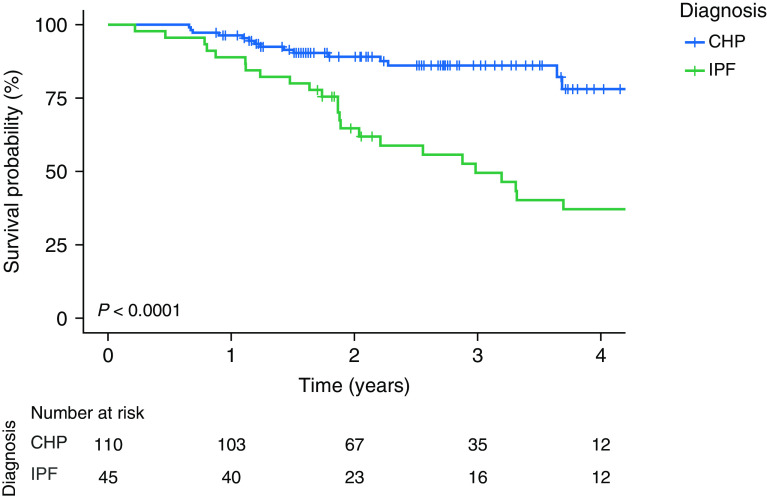
Survival probability of subjects with chronic hypersensitivity pneumonitis (CHP) compared with individuals with idiopathic pulmonary fibrosis (IPF). A Kaplan-Meier curve generated by using a Cox proportional hazards model displaying the survival probability (percentage) of subjects with CHP compared with subjects with IPF is shown. Log-rank *P* test values are reported.

The differences in survival between subjects with CHP and IPF were explored using a multivariate Cox regression analysis, and subjects with CHP were found to survive longer (*P* = 0.0001) than individuals with IPF, even after adjustment of confounding factors: age, sex, smoking history, baseline % predicted FVC, and percentage of honeycombing (Figure 2 and Table E1). Next, using a univariate analysis, we assessed whether there was an association between bacterial burden and survival. No associations were found between bacterial burden and survival in subjects with CHP (hazard ratio, 1.35; 95% confidence interval, 0.92–1.99; *P* = 0.128), whereas burden was associated with mortality in subjects with IPF (hazard ratio, 1.92; 95% confidence interval, 1.32–2.79; *P* = 0.000647). This association remained significant in analysis of IPF using a Cox proportional hazards model adjusted for age, sex, smoking status, and baseline % predicted FVC (hazard ratio, 3.23; 95% confidence interval, 1.64–6.34; *P* < 0.001). This is consistent with published data, which have shown that patients with IPF have an increased pulmonary bacterial load, the magnitude of which predicts the rate of progression of IPF and risk of mortality (16, 18). Collectively, these results suggest that the association between increased bacterial burden and survival is specific to IPF and is not seen in CHP.

### Description of BAL Microbiota Composition in Health and Disease

**Figure 3.**
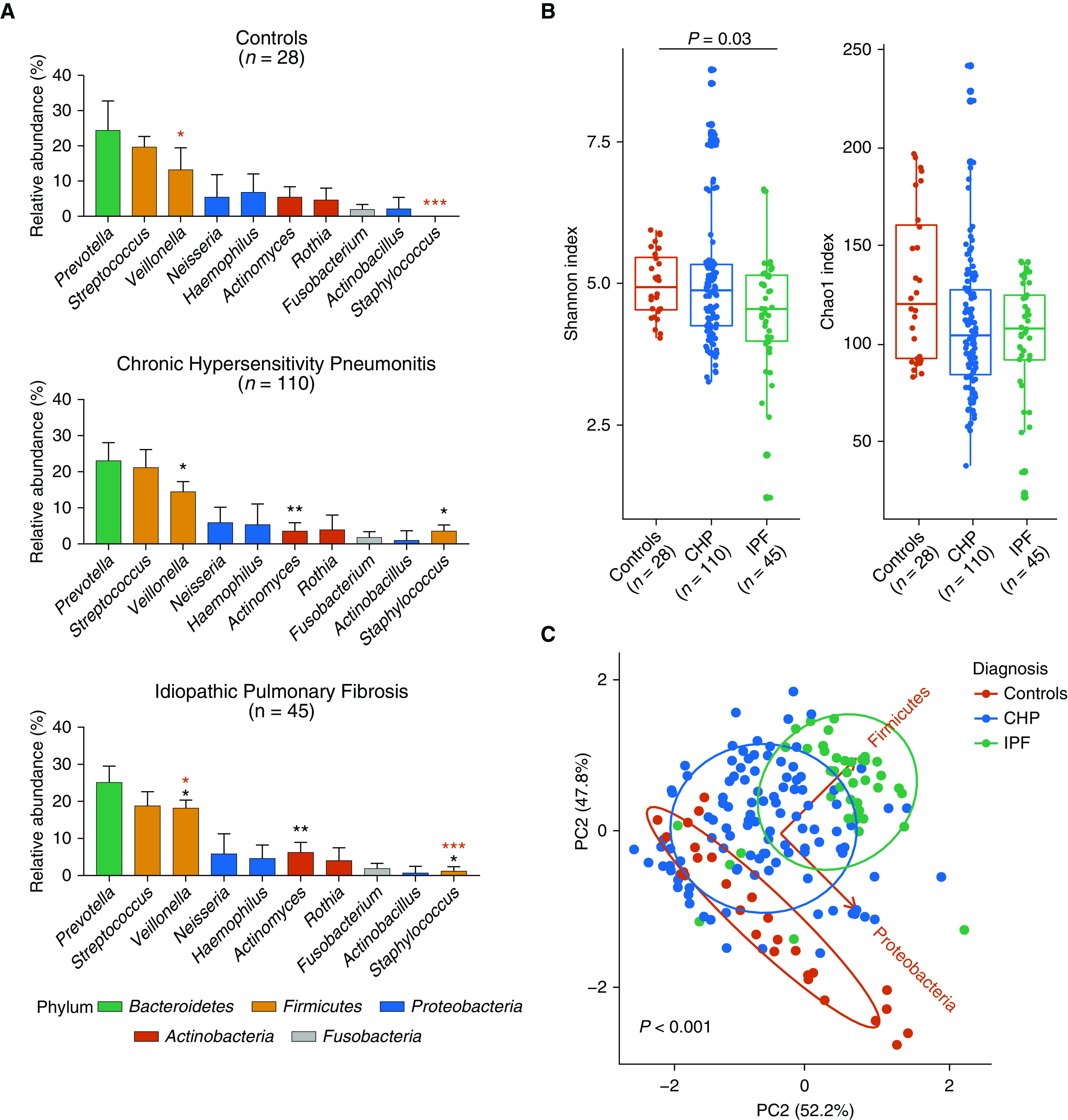
Taxonomic composition of bacteria in BAL of healthy control subjects and subjects with chronic hypersensitivity pneumonitis (CHP) and idiopathic pulmonary fibrosis (IPF). (*A*) Relative abundance of bacteria at the phylum (*Bacteroidetes*, *Firmicutes*, *Proteobacteria*, *Actinobacteria*, and *Fusobacteria*) and genus (*Prevotella*, *Streptococcus*, *Veillonella*, *Neisseria*, *Haemophilus*, *Actinomyces*, *Rothia*, *Fusobacterium*, *Actinobacillus*, and *Staphylococcus*) level. Statistical significance was tested by using the Kruskal-Wallis test with the Dunn multiple-comparison test adjusted with the Bonferroni method. Statistical differences between CHP and IPF are indicated by black asterisks, and those between control subjects and IPF are indicated by red asterisks (*P < 0.05, **P < 0.01, and ***P < 0.001). Data are presented as the mean ± SD. (*B*) Shannon and Chao1 α-diversity measures comparing healthy control subjects and individuals with CHP and IPF diagnoses. Statistical significance was tested by using the Kruskal-Wallis test with the Dunn multiple-comparison test adjusted with the Bonferroni method. Data are presented as the median and interquartile range. (*C*) PC analysis on Euclidean distance comparing healthy control subjects, subjects with CHP, and subjects with IPF (permutational multivariate ANOVA [PERMANOVA], *P* < 0.001). PERMANOVA was adjusted for age, sex, smoking status, FVC (% predicted), and Dl_CO_ (% predicted). PC = principal component.

Across all cohorts, the composition of the microbial communities was dominated by five most abundant phyla: *Firmicutes*, *Bacteroidetes*, *Proteobacteria*, *Actinobacteria*, and *Fusobacteria* (Figure 3A). At the phylum level, the microbiota of subjects with CHP was dominated by *Firmicutes* (38%), *Bacteroidetes* (24%), *Proteobacteria* (23%), and *Actinobacteria* (9%). Although the same phyla predominated in the subjects with IPF, there were a number of differences. In IPF, *Firmicutes* accounted for over 43% of the total reads, and the percentage of reads assigned to *Proteobacteria* was significantly lower compared with the percentage assigned in subjects with CHP (15% and 23%, respectively; *P* *=* 0.04). No significant differences were observed when comparing individuals with CHP with control subjects (Figure E3); in contrast, an increased abundance of *Firmicutes* (43%; *P* = 0.04) and *Actinobacteria* (11%; *P* = 0.04) was noted in subjects with IPF compared with control subjects (Figure E3). Comparison at the genus level identified that the microbial landscape of all groups was dominated by *Prevotella* (control subjects, 24%; subjects with CHP, 23%; subjects with IPF, 25%), *Veillonella* (control subjects, 13%; subjects with CHP, 14%; subjects with IPF, 18%), and *Streptococcus* (control subjects, 19%; subjects with CHP, 21%; subjects with IPF, 18%) (Figure 3A). Of these genera, *Veillonella* (18%) was found to be most abundant in the BAL cells of subjects with IPF compared with the BAL cells of control subjects (13%; *P* = 0.02; Figure E4) and subjects with CHP (14%; *P* = 0.02). Within the *Actinobacteria* phylum, there was a lower relative abundance of *Actinomyces* (4%) in subjects with CHP compared with individuals with IPF (6%; *P* = 0.001) but not compared with control subjects (5%; *P* = 0.95; Figure E4). *Staphylococcus* was found to be increased in subjects with an IPF diagnosis (0.5%) compared with control subjects (0.1%; *P* = 0.03; Figure E4) but was most abundant in the CHP cohort (2%; *P* = 0.0005, compared with IPF cohort) (Figure 3A). Given the previous association between disease progression and an increased relative abundance of *Streptococcus* and *Staphylococcus* in IPF, we investigated whether these bacterial genera were associated with an increased risk of mortality in CHP and IPF. Using a univariate analysis, we found that both *Streptococcus* (hazard ratio, 2.07; 95% confidence interval, 0.83–5.16; *P* = 0.030) and *Staphylococcus* (hazard ratio, 1.80; 95% confidence interval, 0.79–4.09; *P* = 0.033) were significantly associated with increased mortality in IPF. However, consistent with the literature (17), the enrichment of these two bacteria was observed in less than half of the cohort; therefore, it remains unlikely that these organisms alone can explain the disease pathogenesis or progression. Repeating the same analysis in subjects with CHP, no associations between an increased abundance of either *Streptococcus* (hazard ratio, 1.04; 95% confidence interval, 0.34–3.19; *P* = 0.9) or *Staphylococcus* (hazard ratio, 0.25; 95% confidence interval, 0.17–1.59; *P* = 0.3) in the BAL cells of these individuals and disease progression were demonstrated (Figure E5). α-Diversity metrics were used to characterize the distribution (evenness) and number (richness) of taxa expected within each sample. The microbial communities of subjects with IPF had a decreased evenness compared with those of the control subjects (*P* < 0.03) but not compared with those of subjects with CHP. There were no differences in evenness when comparing control subjects with individuals with CHP. Similarly, there were no demonstrable changes when comparing microbial richness among the groups (Figure 3B). Changes in the community composition (β diversity) between healthy control subjects and subjects with CHP and IPF were investigated using PCA and were tested by permutational multivariate ANOVA adjusted for age, sex, smoking status, FVC (% predicted) and Dl_CO_ (% predicted), which confirmed differences in the microbial profiles of the subjects on the basis of diagnosis (*P* = 0.001) (Figure 3C). The PCA revealed that variation among the groups was mostly driven by changes in the *Proteobacteria* and *Firmicutes* phyla, with a tendency toward an enrichment of *Firmicutes* in the BAL from patients with IPF and of *Proteobacteria* in the BAL cells from patients with CHP. Community composition changes among the groups were further interrogated by looking for differentially abundant bacteria using the linear discriminant analysis effect size. Multiple taxonomic differences were found: the BAL fluid of subjects with IPF was enriched with bacteria belonging to the *Firmicutes* phylum, and the BAL fluid of the group with CHP was enriched with bacteria belonging to the *Actinobacteria* and *Fusobacteria* phyla. In contrast, those enriched in the healthy control group belonged to the *Bacteroidetes* phylum (Figure E6). Analyzing the CHP cohort on the basis of antigen positivity or negativity identified no differences between bacterial burden when comparing subjects with CHP with or without an exposure history to an antigen (Figure E7A). Similarly, no differences were observed in the microbial community composition (Figure E7B).

## Discussion

There are clear differences in the composition of the lower airway respiratory microbiota in CHP compared with IPF. Although individuals with CHP have a higher bacterial load in BAL fluid compared with healthy control subjects, it remains significantly lower than that observed in subjects with IPF. In contrast to the mounting evidence for the role of the bacterial burden in IPF, no association was found between the bacterial burden and survival in subjects with CHP.

Over the past decade, studies have begun to unravel the causes and consequences of variation within the respiratory microbiota, improving our understanding of how it may be linked to the pathogenesis of lung diseases. Considerable advances in culture-independent technologies have not only enabled researchers to characterize niche-specific microbial ecosystems but have also enabled them to study the role of the microbiome in lung health and disease (10, 11). Although the spotlight in respiratory research has been on asthma, COPD, and IPF, CHP remains an understudied disease (12, 13, 15–18). Research into the lung microbiota in CHP is very limited; to date, no specific study has investigated the composition of microbial communities in the lower airways in CHP.

The baseline bacterial phyla that dominated the BAL fluid of control subjects and subjects with CHP and IPF in the present study were *Firmicutes*, *Bacteroidetes*, *Proteobacteria*, *Actinobacteria*, and *Fusobacteria*, which have previously been reported to be present in the airways of healthy subjects and patients with asthma, COPD, and IPF (10, 36). Marked differences were evident in the intergroup distribution of phyla. Specifically, an increased abundance of *Firmicutes* and *Actinobacteria* was noted in subjects with IPF compared with control subjects and subjects with CHP. In contrast, individuals with CHP had an increased proportion of *Proteobacteria* compared with individuals with IPF. This finding is consistent with the results of a recent study of subjects with IPF who had an increase in *Firmicutes* and a decrease in *Proteobacteria*; this reduction in microbial diversity was associated with disease activity in subjects with IPF (36). Furthermore, studies have shown that during respiratory disease, there is an increased prevalence of *Firmicutes* and *Proteobacteria* (12). This observation suggests that an altered state of the lung microbiome may be directly associated with the pathophysiology of fibrotic lung disease.

At the genus level, the microbial profile of all groups was characterized by *Prevotella*, *Veillonella*, and *Streptococcus*, bacteria known to prevail and/or dominate in the airways of healthy subjects and patients with respiratory disease (11). The phylum *Firmicutes* is associated with an increased risk of IPF progression when found at increased relative abundance in the lung (17). Specifically, within the *Firmicutes* phylum an increased abundance of *Streptococcus* and/or *Staphylococcus* species has been shown to be strongly correlated with reduced progression-free survival time in IPF (17). In the present study, *Staphylococcus* was increased in subjects with IPF compared with healthy control subjects and was further increased in subjects with CHP compared with subjects with IPF. In addition, in agreement with previous findings, *Veillonella* was also found to be increased in subjects with IPF compared with control subjects (16) and subjects with CHP. We further delineated differences in microbial evenness and whole community composition between CHP and IPF. Collectively, these findings show that subjects with CHP have a distinct microbiome compared with patients with IPF and healthy control subjects, which suggest that alterations in the microbiome characterized by an increased abundance of bacteria belonging to the *Firmicutes* phylum may play a role in the pathogenesis of these interstitial lung diseases. However, we cannot as yet draw any causal conclusions about this altered respiratory microbiome in CHP ahead of functional and longitudinal studies to comprehensively examine pathogenic host–microbe interactions.

It has previously been shown that IPF is characterized by an increased bacterial burden in BAL fluid compared with control subjects and that the bacterial load at the time of diagnosis predicts rapidly progressive IPF and an increased risk of mortality (16, 17). Given the radiological overlap and similarities in clinical behavior between patients with CHP and patients with IPF, it was surprising to find such striking differences in the lower airway bacterial burden between these two groups of patients. Although the BAL bacterial load in subjects with CHP was higher compared with that in control subjects, it was still lower than that in patients with IPF. Comparable with authors of other studies, we validate the finding that a higher bacterial burden at the time of diagnosis predicts subsequent progression of IPF. This association was not evident in the group with CHP and may explain why immunosuppression has been shown to be harmful in IPF but remains widely used to treat CHP in the absence of similar observations (37). Regardless of bacterial burden, subjects with CHP survived longer than individuals with IPF, a result that mirrors the findings of Vasakova and colleagues (38).

Closely associated with the alterations in the microbial composition is the change in the host immune status of patients with fibrotic lung disease. Specifically, lymphocytes, which are markedly increased in the airways of patients with CHP, play a key role in the inflammatory process underpinning the pathogenesis of the condition (39). An increased proportion of BAL lymphocytes was also present in the subjects with CHP in the present study. However, in agreement with what has been previously shown in IPF (16), no associations were found between BAL differential leukocyte counts and the bacterial burden in these subjects. It is increasingly recognized that a reciprocal communication likely exists between the microbiome and the pulmonary immune system, which may be critical for promoting pathology in a number of respiratory conditions (40). Future elucidation of microbial composition as well as the immune-cell subsets involved will improve our understanding of the cross-talk between microbiome and host.

There are a number of limitations to our work. Procedural and sequencing contamination presents a critical problem when evaluating samples with low microbial biomass, such as samples from the lungs (41). To minimize the risk of contamination, low-biomass protocols for DNA extraction were used, and negative bronchoscopy control subjects and reagent control subjects were sequenced alongside the BAL samples. The inclusion of the negative control samples, which yielded a bacterial burden close to or below the lower limit of qPCR quantification (100 copies/ml of BAL fluid) and had a taxonomic profile distinct from that of the BAL specimens, robustly indicates that the sequencing protocol used resulted in no apparent contamination. Furthermore, samples from case patients and control subjects were always processed in a blinded, randomized fashion to uphold identical storage and processing conditions across all samples. Another significant limitation is that 16S rRNA gene sequencing provides a snapshot of the microbial community at a particular moment in time, and it is therefore challenging to draw conclusions regarding whether the altered microbial community is a cause or a consequence of the disease (42). Finally, this exploratory study was based on feasibility of recruitment and sampling, and some of the most severe cases of both CHP and IPF may have not been included, as only subjects able to safely undergo bronchoscopy were enrolled. Therefore, functional and longitudinal studies examining host–microbe interactions are needed to validate these findings.

In conclusion, in this prospective observational cohort study, we provide the first evidence of the composition of the microbial communities in the lower airways in CHP. The microbial profile of subjects with CHP is distinct from that of subjects with IPF. Compared with subjects with IPF, these individuals have a decreased bacterial burden, which does not predict survival. Future work should aim at understanding whether the total bacterial load and relative abundances of the altered microbial genera may be acting synergistically to advance the fibrotic process.
